# ddRAD-seq reveals the genetic structure and detects signals of selection in Italian brown trout

**DOI:** 10.1186/s12711-022-00698-7

**Published:** 2022-01-31

**Authors:** Gabriele Magris, Fabio Marroni, Edo D’Agaro, Massimo Vischi, Cristina Chiabà, Davide Scaglione, James Kijas, Maria Messina, Emilio Tibaldi, Michele Morgante

**Affiliations:** 1 Department of Agricultural, Food, Environmental and Animal Sciences (Di4A), University of Udine, Via delle Scienze 206, 33100 Udine, Italy; 2 Istituto di Genomica Applicata, Via J. Linussio 51, 33100 Udine, Italy; 3 IGA Technology Services, s.r.l, Via J. Linussio 51, 33100 Udine, Italy; 4 CSIRO, 306 Carmody Road, St Lucia, QLD 4067 Australia

## Abstract

**Background:**

Brown trout is one of the most widespread fresh-water fish species in Europe. The evolutionary history of and phylogenetic relationships between brown trout populations are complex, and this is especially true for Italian populations, which are heavily influenced in different ways by stocking practices. The characterization of the genetic structure of Italian brown trout populations may give information on the risk of losing endemic Italian populations due to lack of genetic diversity or to admixture with stocking populations. The identification of signatures of selection, and the information deriving from dense genotyping data will help genotype-informed breeding programs. We used a ddRAD-seq approach to obtain more than 100,000 single nucleotide polymorphisms (SNPs), and to characterize the population structure and signatures of selection in 90 brown trout samples.

**Results:**

Italian brown trout populations are genetically differentiated, although the stocking practices have introduced strong admixture in endemic Italian trout, especially with the Atlantic lineage. Most of the analysed populations showed high levels of kinship and inbreeding. We detected putative signatures of selection using different approaches, and investigated if the regions were enriched for functional categories. Several regions putatively under selection and characterized by a reduction in heterozygosity across all the studied populations are enriched for genes involved in the response to viral infections.

**Conclusions:**

Our results, which show evidence of admixture with the Atlantic lineage (commonly used for stocking), confirm the need for controlling stocking practices, in order to avoid the erosion of the endemic gene pool; given the apparently high levels of kinship and inbreeding in local populations, our results also show the need to take action for increasing gene diversity. In addition, we used the genetically-distinct lineages to detect signatures of selection and we identified putative signatures of selection in several regions associated with resistance to infectious diseases. These constitute candidate regions for the study of resistance to infections in wild and farmed trout.

**Supplementary Information:**

The online version contains supplementary material available at 10.1186/s12711-022-00698-7.

## Background

Brown trout is among the most widespread fresh-water fish species in Italy and in Europe, and is characterized by a high phenotypic and genetic variation throughout its natural distribution range [1]. The evolutionary history of brown trout and the phylogenetic relationships among trout populations are complex; as a result, the systematic status of Italian brown trout is still a matter of controversies [2]. In spite of the commercial [3] and ecological relevance of Italian brown trout [4], its genetic composition is still not fully understood. While there is general agreement that the majority of brown trout specimens should be attributed to the *Salmo trutta* species, the same morphological variants are considered as species or subspecies by different authors [5] according to the splitter-lumper dichotomy often observed in phylogenetics [6]. To cite some examples, marble trout has often been presented as an independent species, *Salmo marmoratus* [7–10] and sometimes as a subspecies of *S. trutta* [2, 6]. A similar situation is observed for the Garda’s carpione, which again can be regarded as an independent species, *S. carpio* [7, 9] or as a subspecies of *S. trutta* [2]. Analysis of the mtDNA of the Garda’s carpione revealed the presence of haplotypes that are shared with other lineages, such as the Marmoratus, which led researchers to conclude that the Garda’s carpione originated by introgression from those lineages [11]. Regardless of the real phylogenetic structure, some researchers believe that studies that are performed by picking previously described species one by one only should be avoided if the aim is to gain further insight into the genetic structure of trout [6]. A more detailed understanding of the genetic structure of brown trout populations may be useful for conservation practices. However, disentangling the genetic structure of brown trout is hampered by the complicated phylogenetic structure of trout populations in Italy [2, 9], and by the repeated hybridization events between highly diversified populations, sometimes considered as different species [8]. According to previous studies, gene flow between brown trout and marble trout has been extensively reported, while the Garda’s carpione seems to be still genetically isolated from other Salmo individuals [7]. In addition, other studies have reported a clear differentiation between *Salmo trutta fario* and *Salmo marmoratus*, and a less clear differentiation of these two from *Salmo carpio* [8]. Introgression with non-native species and/or populations and with hatchery samples also contributes to the complicated genetic structure and endangers the stock of the native brown trout populations [4]. The practice of stocking with hatchery samples probably affects the vast majority of Italian water-courses [12]. Gene flow from non-native species is only one of the possible threats to brown trout populations. Their small population size can lead to high levels of relatedness and inbreeding, thus eroding their genetic diversity and possibly leading to the extinction of local trout lineages.

Genetic structure may also interact with environmental factors in determining trout phenotypes, such as resistance to pathogens [13]. Previous studies identified signatures of selection in genes that are postulated to affect migration tendency [14, 15], immunity [16], and feeding behavior [17] in European brown trout populations. In addition, studies based on short tandem repeats (STR) were able to identify signatures of selection related to population structure in brown trout populations from Northern Europe [18, 19]. This prompted us to investigate signatures of selection in genes that have contributed to the differentiation (or lack of it) between Italian brown trout lineages. While the study of signatures of selection may be focused on candidate loci with a relatively small number of markers, the genome-wide detection of signatures of selection is more efficient with a large number of genetic markers [20].

Most of the previous studies that have investigated the evolutionary history of Italian brown trout have used relatively small sets of DNA markers [2, 9]. Only one study used double digest restriction-site associated sequencing (ddRAD-seq) to genotype a large number of markers [1]; a follow-up study using the same samples also revealed that stocking practices had an impact on population structure [21]. ddRAD-seq is an inexpensive approach to generate high-density genotyping datasets in model and non-model organisms. Unlike fixed content platforms such as single nucleotide polymorphism (SNP) arrays, ddRAD-seq datasets are free from the ascertainment bias that can affect studies that rely on genotyping data derived using SNP arrays [22]. Thus, we used a ddRAD-seq approach to genotype more than 100,000 SNPs in a diverse panel of brown trouts, to investigate the genetic structure of Italian trout populations and detect signatures of selection. Given the long lasting difficulties in clearly defining the phylogenetic relationships between Italian brown trout populations, we identified population clusters based on genetic data, and we use the generic term “lineages” to refer to them without any implication regarding their taxonomic rank.

## Methods

### Sample collection

Samples were collected from several regions in Italy, from Corsica, and from Austria. Approximately half of the individuals were collected from fish farms and half from rivers. The collected samples belong to several lineages that have already been described in Italy and show distinct phenotypic characteristics, namely Marmoratus, Carpione, Atlantic and Mediterranea. The extensive phenotypic differentiation between Marmoratus and Carpione has led some authors to consider them as distinct species (*Salmo marmoratus* and *Salmo carpio*, respectively) [8]. In the present study, we did not attempt to disentangle the complex phylogeny of brown trout, and used the generic term ‘lineages’ to refer to distinct phenotypes. A detailed list with all available information on the origin of samples is in Additional file 1: Table S1.

### DNA extraction, library preparation and sequencing

DNA was extracted from dorsal fins, muscle or scales (depending on availability) using the MagAttract HMW DNA kit (Qiagen, Hilden, Germany). ddRAD libraries were prepared and sequenced by IGA technology services s.r.l (Udine, Italy), using a custom protocol after minor modifications to the original ddRAD protocol [23]. Briefly, genomic DNA was fluorimetrically quantified, normalized to a uniform concentration and double-digested with the SphI and BstYI enzymes. Fragmented DNA was purified by using AMPureXP beads (Agencourt) and ligated to barcoded adapters. Samples were pooled on multiplexing batches and bead-purified. For each pool, the BluePippin instrument (Sage Science Inc.) collected distributions of targeted fragments. Each gel eluted fraction was amplified with oligo primers that introduce TruSeq indexes and subsequently bead-purified. The resulting libraries were checked both on a Qubit 2.0 Fluorometer (Invitrogen, Carlsbad, CA) and by a Bioanalyzer DNA assay (Agilent technologies, Santa Clara, CA). Libraries were processed with the Illumina cBot system for cluster generation on the flow cell following the manufacturer’s instructions, and sequenced using the V4 chemistry and the paired-end 2 $\times$ 125 bp mode on a HiSeq2500 instrument (Illumina, San Diego, CA).

### Alignment and variant calling

Bioinformatic analysis from raw reads to genotypes was performed by IGA technology services s.r.l. (Udine, Italy). Briefly, Illumina reads were demultiplexed using the process_radtags utility included in the Stacks v2.0 software [24]. Alignment to the *Salmo trutta* v1.1 reference genome (NCBI accession: PRJEB32115) was obtained using the BWA-MEM algorithm [25] with default parameters and selection of uniquely aligned reads (i.e. reads with a mapping quality > 4). Detection and genotyping of all the loci from the aligned reads were done by using the gstacks program included in Stacks v2.0 [24]. The detected loci were filtered using the software ‘populations’, which is included in Stacks v2.0 and was run with the following options: –R = 0.75 in order to retain only the loci that are present in at least 75% of the population, and –max-obs-het = 0.8 to remove SNPs that are in the heterozygous state in more than 80% of the samples.

The following filters were also used to ensure high-genotyping quality:

- Exclusion of samples with an average coverage lower than 5× from further analysis, i.e. six samples;
- For each individual, positions at which the coverage was lower than 5× were set to “uninformative”.

Finally, only the positions that were informative in at least 50% of the individuals were retained for further analysis.

### Population analysis

Admixture analysis [26] was used to investigate population structure with the most probable number of populations, K, being determined by cross-validation. For each individual, the admixture algorithm returns the probability Q (Q_1_, Q_2_, …, Q_K_) that it belongs to each of the K populations, and the quantity maxQ is defined as the highest Q for each individual. Subjects with a maxQ higher than 0.95 are considered as admixed. Then, the software NewHybrids [27] was used to further characterize the hybrid structure of the populations based on a subset of SNPs selected with high F_ST_ values (> 0.95) and low linkage disequilibrium (LD) values (< 0.2). PCA analysis was performed using the R [28] package SNPRelate [29]. A maximum likelihood phylogenetic tree [30] with bootstrap estimates was obtained using SNPhylo [31], and drawn with the web tool iTOL [32]. A maximum likelihood phylogenetic tree [30] accounting for migration events was built using Treemix [33]. The number of allowed migration events ranged from 1 to 6, but all the analyses showed at most three gene flow directions, and the analysis with three migration events was chosen for plotting.

The relatedness between individuals (within and between lineages) was assessed using the maximum likelihood estimator of identity-by-descent (IBD) implemented in the R package SNPRelate [29]. To avoid the impact of LD on the IBD estimate, one SNP was removed from each pair of SNPs with an LD value greater than 0.2. Inbreeding coefficients were calculated using the same set of SNPs and the maximum likelihood estimator implemented in SNPRelate [29]. To assess the effect of rare alleles on estimates of relatedness and inbreeding, the analyses were repeated by setting the MAF to values higher than 5, 10, 20 and 25%, respectively. The coefficient of inbreeding F_IS_ was computed as 1-Ho/He, where Ho is the observed heterozygosity, and He is the expected heterozygosity under Hardy–Weinberg equilibrium.

Expected heterozygosity was computed for each SNP in the whole dataset, in the samples of farmed and wild individuals separately, and in samples stratified by population; SNPs with at least 10 genotyped individuals per group were considered informative. Expected heterozygosity corresponds to gene diversity [34], and dividing this value by the number of sequenced bases, gives the nucleotide diversity π.

### Putative signatures of selection

The ZH_p_ statistics [35] is the Z-transformation of the pooled heterozygosity for a selected pool of individuals (H_p_) and we used it to identify putative signatures of selection that result in the enrichment or depletion of homozygosity in pools of individuals. Pools were defined as individuals belonging to each of the five lineages identified by the admixture analysis. The analysis was performed on the whole sample of individuals and separately on each population, which enabled the detection of signatures of selection that are private to each population or shared between populations. The analysis was repeated by removing admixed individuals (individuals with a Qmax < 0.95), to assess their contribution to the signal. In addition, the analysis was repeated by stratifying samples into farmed and wild individuals. All the analyses were conducted using a sliding window approach, with a window length of 1,000,000 bp and a step of 200,000 bp, consisting on average of 67 SNPs. Results with a ZH_p_ score lower than −2.81 or higher than 2.81 (an arbitrary threshold, corresponding to a two-tail p-value ≤ 0.005), were considered significant. Putative regions undergoing selection were defined as those for which at least two windows with a significant ZH_p_ score overlapped. To assess the number of regions that meet this requirement due to chance alone, a resampling experiment was performed by randomly reshuffling window positions 1000 times and measuring the probability of observing any given number of overlapping windows exceeding the Z-score threshold; regions with a p-value lower than 0.05 based on the resampling were retained for further analysis.

The HapFLK software was used to perform haplotype-based analysis of regions undergoing selection [36], setting K (local haplotype cluster to be used as proxy for alleles) to 20, and the number of iterations to 20 (default = 10), to achieve a good compromise between accuracy and computation time [36]. Briefly, HapFLK is an extension of the F_ST_-based analysis in that it detects genomic regions in which haplotype stretches show exceedingly high (or low) divergence between individuals belonging to the studied populations, and takes population structure into account. Thus, HapFLK should be effective in detecting recent selective sweeps, which differentiate the studied populations. The HapFLK software also provides tools to convert the test statistic p-value [36, 37]. Putative regions undergoing selection are defined as those for which a window composed of at least two consecutive SNPs shows a nominal p-value ≤ 0.01. To assess the number of regions that meet this requirement due to chance alone, a resampling experiment was performed by randomly reshuffling SNP positions 1000 times and measuring the probability of observing regions of any given number of consecutive SNPs meeting the above mentioned criteria; regions with a p-value lower than 0.05 based on the resampling were retained for further analysis. Analyses were repeated by stratifying samples into farmed and wild individuals and by excluding individuals with a maxQ lower than 0.95.

A sliding window kinship estimation via IBD was performed using SNPRelate [29], to identify the specific regions that are shared between populations, as a proxy for signatures of selection. Window size was set to 5 Mb with a step of 2.5 Mb. Candidate regions were retained if at least two overlapping windows showed an average kinship coefficient across all pairwise populations comparisons of 0.05 or more. To assess the number of regions that meet this requirement due to chance alone, a resampling experiment was performed by randomly reshuffling window positions 1000 times and measuring the probability of observing any given number of overlapping windows exceeding the kinship coefficient of 0.05; regions with a p-value lower than 0.05 based on the resampling were retained for further analysis. Analyses were repeated by stratifying samples into farmed and wild individuals and by excluding individuals with a maxQ lower than 0.95.

Recombination rates in the regions close to putative regions under selection were calculated. A high-density linkage map for *Salmo trutta*, as well as the recombination rates and sequence tags that were used to anchor it on the *Salmo salar* genome, are available [38]. These sequence tags were used to anchor the linkage map to the *Salmo trutta* genome using the blast tool [39]. To avoid spurious mapping, only the hits with an e-value lower than 10^–30^ and a percentage of identity higher than 95% were retained. The recombination rates estimated on the *Salmo salar* genome were used as proxies for those on the *Salmo trutta* genome. The recombination rates within the regions surrounding the putative selected regions were computed as the average recombination rate in the selected region and that within the regions 1 or 5 Mb upstream and downstream. These 1- and 5-Mb distances were selected to have a local measure of recombination rate and to increase the number of windows for which an approximate estimate of recombination rate was available, respectively. If none of the markers on the linkage map was anchored near the putative selected region, the result was set to Not Available (NA).

### Functional annotation

Functional annotation of *Salmo trutta* v1.1 reference genome transcripts as gene ontology (GO) and Kyoto Encyclopedia of Genes and Genomes (KEGG) terms was performed based on the following workflow:

1. Transcripts were aligned to the uniprot database using the blastx tool [39], with an Expect Value (e-value) threshold of 10^–10^; mapping transcripts were associated to one or more Uniprot ID.
2. The Uniprot match of each transcript was used as a search term against the GO database downloaded from the Gene Ontology Annotation project website [40]. Matching Uniprot terms were associated to one or more GO term.
3. Using the KEGGREST package (http://bioconductor.org/packages/release/bioc/html/KEGGREST.html), the Uniprot terms obtained in step (1) were used as search terms against the full KEGG database. Each matching gene id was associated to one or more KEGG term.
4. Using the KEGG name as a search term, KEGG class and KEGG pathway were extracted from the KEGG database.

As a result of the annotation, each transcript was associated to zero, one, or several Uniprot entries, GO terms, KEGG class and KEGG pathway (map); the resulting file is publicly available.

Enrichment analysis was performed based on KEGG pathways (generally representing broader biological categories and thus including more genes). Enrichment was tested in each pathway only if the number of occurrences of the term was greater than 3; only significant results (p-value < 0.05) are shown.

## Results

### Animal genotyping

**Fig. 1:**
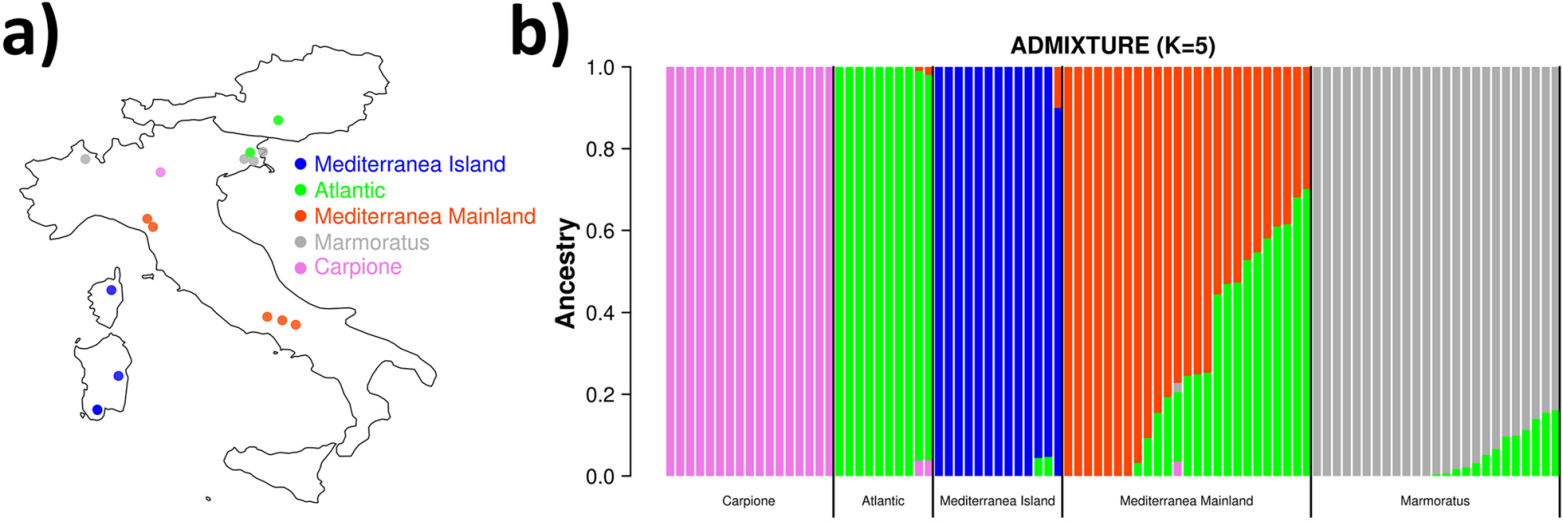
Geographic (**a**) and genetic (**b**) structure of the studied sample. **a** Sampling locations. Colored points on the map represent sampling locations. **b** Admixture analysis. Each barplot represent an individual. The height of each colored bar represents the probability of assignment to the corresponding ancestry, when assuming the presence of five ancestral populations (K = 5)

Ninety-six fishes were sampled from several regions in Italy, from Corsica and from Austria (see Additional file 1: Table S1) and Fig. 1a. This set included both farmed and wild caught fish that represent the previously identified lineages of Marmoratus, Carpione, Atlantic and Mediterranea (see Additional file 1: Table S1). A summary of the studied populations and their composition is in Table 1. The use of farmed and wild caught animals is probably not ideal, and was driven by sample availability; due to the common practice of stocking with domesticated samples [2, 4], this approach has already been applied [8], and is somehow representative of the large impact that farming practices may have on the Italian trout gene pool. However, no detailed information on farming history and intensity is available. Based on the results discussed below, in the current study, the Mediterranea region was further divided into Mediterranea Island and Mediterranea Mainland (Fig. 1b) and (see Additional file 1: Table S1), resulting in five lineages. In some parts of this paper, its figures and tables, lineages are indicated by their initials, as follows: AT = Atlantic, CA = Carpione, MA = Marmoratus, MI = Mediterranea Island, and MM = Mediterranea Mainland.

**Table 1:** Composition of the studied samples

Lineage	Code	Farmed	River	Unknown	N Admixed	Avg maxQ	π
Atlantic	AT	10	0	0	1	0.989	0.43 × 10^–3^
Carpione	CA	17	0	0	0	1.000	0.28 × 10^–3^
Marmoratus	MA	22	0	3	8	0.962	0.74 × 10^–3^
Mediterranea island	MI	0	13	0	1	0.985	0.33 × 10^–3^
Mediterranea mainland	MM	0	25	0	17	0.777	1.1 × 10^–3^

*Farmed* number of samples collected in fish farms, *River* number of samples collected in rivers, *Unknown* number of samples for which no origin information was provided, *N admixed* number of admixed individuals (maxQ < 0.95), *Avg maxQ* average value of maxQ in the population, **π** nucleotide diversity

To obtain high-density SNP genotypes, 226,406,883 reads were generated and successfully demultiplexed. The number of demultiplexed reads per sample ranged from 20,578 (sample MI13) to 4,624,216 (sample MM04). Average coverage ranged from 2.46 × (MA20) to 42.29 × (MM04) with a median of 14.39. The number of demultiplexed reads per sample and the corresponding average coverage are listed in Additional file 1: Table S1. Six samples with an average coverage lower than 5× were removed from further analysis. In total, 126,124 SNPs that passed the filtering criteria and were distributed across the 40 *Salmo trutta* chromosomes were retained for further analysis. The average number of SNPs per chromosome was 3153, ranging from 1215 (chromosome 11) to 5087 (chromosome 14). A full list of the number of SNPs per chromosome is in Additional file 2: Table S2. Inbreeding and kinship analyses were repeated by varying MAF. Removing the SNPs with a MAF higher than a given threshold and (as required for inbreeding and kinship analysis) the SNPs showing an LD value with another SNP higher than 0.2 decreased the number of SNPs used in the subsequent analyses. For example, 16,282 SNPs were retained for inbreeding analysis (LD lower than 0.2) (see Additional file 3: Table S3).

### Population structure

To explore the degree of population substructure and admixture between populations, we performed cross-validation of admixture analysis and obtained an optimal number of K = 5 clusters (See Additional file 4: Fig. S1). Figure 1b shows that the five clusters clearly separated the four previously identified lineages (Marmoratus, Carpione, Atlantic and Mediterranea) and further separated the Mediterranea collected in Sardinia and Corsica (Mediterranea Island) from the Mediterranea samples collected in several locations from mainland Italy (Mediterranea Mainland).

All lineages were separated from each other, although some admixture between Mediterranea Mainland and Atlantic was observed. This reflects the high differentiation among the trout populations observed in Italy, and at the same time, confirms the long-lasting history of introduction of Atlantic individuals into central Italy for stocking [2, 5]. Also, the presence of an Atlantic gene pool in some Marmoratus individuals may result from the stocking of rivers inhabited by Marmoratus with Atlantic individuals, as previously reported [5]; this risk of stocking practices for endemic lineages was mentioned more than 20 years ago [8]. The results presented here provide clear evidence of the admixture of Atlantic strains into other populations, and in the case of the Mediterranea Mainland population investigated here, we found that the proportion of the contribution from Atlantic ancestry exceeded 50% in some fish. Mediterranea Island samples may represent a morph that is sometimes classified as *Salmo macrostigma* (or *S. cettii*, [41]) commonly found in Sicily, Sardinia, Corsica and southern Latium [2], but also in North Africa, which would explain why the two regions Mediterranea Island and Mediterranea Mainland are very different. Individual ancestry coefficients estimated by admixture analysis are reported in Additional file 1: Table S1, together with the maxQ values, i.e. the highest attribution probability for each individual. When maxQ is lower than 1, the individual can be regarded as admixed; maxQ values lower than 0.95 are highlighted in Additional file 1: Table S1. Among all the samples, those from the Mediterranean mainland population have the highest levels of admixture. In particular, the subpopulations sampled in Tuscany (MM11 to MM15) and Emilia-Romagna (MM16 to MM20) showed high levels of admixture with Atlantica, and only the subpopulation sampled in Campania (MM21 to MM25) showed no admixture with Atlantica. These results support the idea that the long-lasting practice of stocking rivers in the Apennine regions with trouts from the Atlantic lineage has led to substantial erosion of the endemic gene pool [2, 4].

In addition to admixture analysis, we performed principal component analysis (PCA) to explore the clustering of individuals using the same SNP dataset (see Additional file 5: Fig. S2), which revealed a similar scenario, with the five groups being clearly separated. The Mediterranea Mainland and Atlantic clusters did not overlap, but a number of individuals formed a cluster in close proximity, which supports the results of admixture analysis (Fig. 1).

**Fig. 2:**
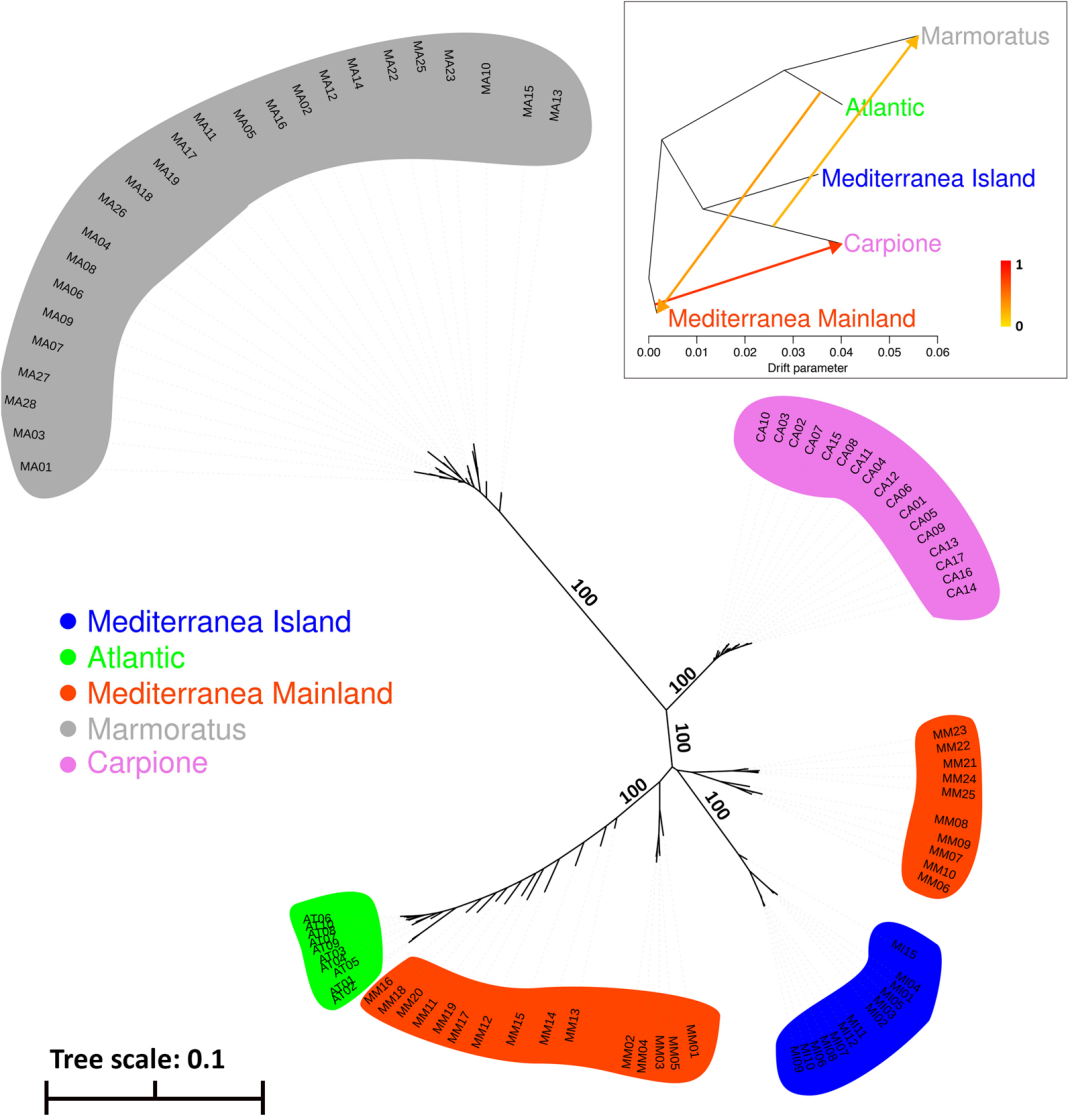
Maximum likelihood phylogenetic tree and phylogenetic tree with migration events inferred by Treemix (inset). Lineages were assigned based on the results of the Admixture analysis shown in Fig. 1a. In the inset, inferred migrations between populations are shown as arrows

Figure 2 shows the phylogenetic tree of the studied samples. The five populations are clearly separated, with Marmoratus being the most distantly related to the others, in agreement with reports that place Marmoratus as a different species, *S. marmoratus* [8, 9]. One interesting feature of the tree is the separation of the Mediterranea Mainland individuals into two main clusters; one to the right and one to the left of Mediterranea Island, with the latter composed by several individuals that were not clearly separated from the Atlantic lineage. Individuals in the cluster to the left of Mediterranea Island are the most admixed members of the Mediterranea mainland samples, originating from the Biferno, Secchia, and Serchio rivers, with an average maxQ value of 0.653 and for which the other admixed population component belonged to Atlantic (Fig. 1) and (see Additional file 1: Table S1). The predicted Mediterranea Mainland individuals that are in the cluster to the right of Mediterranea Island are those sampled in the Fibreno and Volturno rivers, with an average maxQ of 0.963, thus representing the less admixed components of the Mediterranea Mainland individuals. Gene flow between Atlantic and Mediterranea Mainland was also identified by Treemix and is shown by an arrow connecting the two populations in the inset of Fig. 2.

### Gene flow between lineages

We used several approaches to understand past and present gene flow events between lineages. Additional file 6: Table S4 shows the results of the analysis with the NewHybrids software that was performed on the individuals showing some degree of admixture. This NewHybrids analysis considered that eight of the samples belonged to the parental population (i.e. that are not hybrids) with a posterior probability greater than 80%, but lower than 90%. These eight samples showed the lowest degree of admixture (0.7 < maxQ < 0.95), and it was difficult to decide if they are still representative of the endemic gene pool or if they are substantially admixed. For the remaining 10 individuals, a higher level of admixture (0.5 < maxQ < 0.71) was observed, which indicates an approximately equal probability of belonging to the Atlantic and Mediterranea mainland groups. The NewHybrids analysis also clearly indicated an F2 kind of hybridization, with probabilities greater than 0.98. This suggests that the hybridization event is relatively recent and that the proportion of alleles derived from the farmed individuals is high in river samples (see Additional file 6: Table S4).

In addition, we performed a phylogenetic analysis to build a phylogenetic tree and, at the same time, to infer gene flow events between populations using Treemix (Fig. 2, inset). Gene flow events are shown as arrows, and they suggest migration from Mediterranea Mainland to Carpione, from Atlantic to Mediterranea Mainland, and from Carpione to Marmoratus.

The gene flow events between the Atlantic and Mediterranea Mainland groups that are revealed in our admixture analysis are well known and have already been documented [2, 5]. Gene flow between the Carpione and both the Mediterranea Mainland and Marmoratus groups has also been previously detected and explained by hypothesizing that Carpione originated from genetic contributions from the Mediterranea and, to a lesser extent, from the Marmoratus lineages [9]. Giuffra et al. [8] reported a similar result by studying the mtDNA and the protein sequences of fishes sampled in the river Po, and suggested that *S. carpio* (Carpione, in our study) originated from the hybridization between *S. marmoratus* (Marmoratus) and *S. trutta fario* (Mediterranea Mainland) [8]. This scenario fits relatively well with our Treemix results, although apparently they support a contribution of Carpione to Marmoratus, rather than the opposite direction. However, we found no significant evidence of admixture between Carpione and Marmoratus or between Carpione and Mediterranea Mainland in our admixture analysis, and the PCA analysis (see Additional file 5: Fig. S2) clearly separated the three samples from each other.

### Diversity within populations

**Fig. 3:**
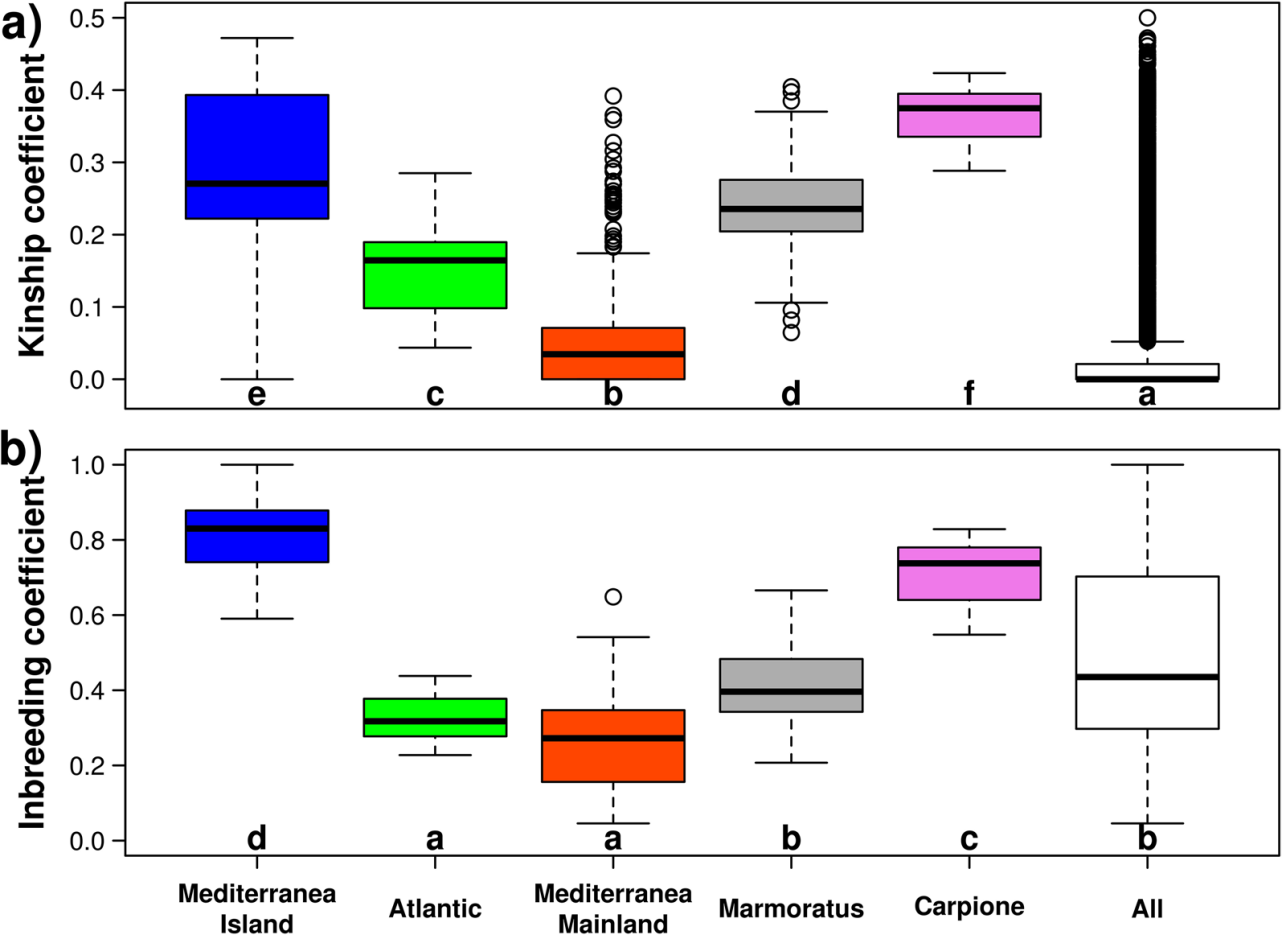
Distribution of kinship (**a**) and inbreeding coefficients (**b**). All: statistics computed from the complete dataset considered as a single population. Samples with the same letter are not significantly different from each other according to Wilcoxon’s test. Boxes indicate the first and third quartiles, the horizontal line within the boxes indicates the median and the whiskers indicate ± 1.5× interquartile range

The distribution of kinship coefficients in all pairwise comparisons between individuals of the same lineage (colored boxplots) and in all pairwise comparisons irrespective of the population of origin (white boxplot) are shown in Fig. 3a. Generally, the levels of relatedness between individuals were very high, with only the Mediterranean Mainland population showing a median kinship coefficient lower than 0.25. Carpione showed the highest median level of kinship coefficient, and this is in agreement with the very dense cluster that the Carpione samples formed in the PCA analysis (see Additional file 5: Fig. S2). Very high relatedness between trout individuals has been previously observed in small tributary populations [42], or in populations of individuals originating from a single location [43]. The median kinship coefficient between all pairwise comparisons irrespective of the population of origin is much lower than the within-population kinship coefficient, which testifies that high levels of relatedness are mostly observed in local populations.

Figure 3b shows the estimated inbreeding coefficients of the populations, with again very high levels of inbreeding, the Mediterranea Island and Mediterranea Mainland populations showing the highest and lowest levels of inbreeding, respectively.

Previous studies based on a similar number of SNPs obtained comparable (although lower) levels of inbreeding, with samples from hatcheries showing generally higher levels than the wild samples [1]. In our study, both the highest (Mediterranea Island) and lowest (Mediterranea Mainland) levels of inbreeding were observed in wild populations. The inbreeding coefficient estimated on the whole population is obtained by considering the samples as originating from one population. For the sake of comparison, we also computed F_IS_ for the whole sample, and again we obtained a strong evidence for inbreeding, with an average F_IS_ of 0.39.

Since the estimation of the kinship and inbreeding coefficients may be affected by the presence or absence of rare alleles, we repeated the analysis by increasing the MAF required for including SNPs in the study (see Additional file 7: Fig. S3), and observed that our results are stable across all tested MAF thresholds.

A closer look at the Mediterranea Mainland population stratified by river of origin (see Additional file 8: Fig. S4) revealed that the samples with the highest degrees of admixture (those originating from the Serchio and Secchia rivers, (see Additional file 1: Table S1) are those with the lowest inbreeding and kinship coefficients. This raises an interesting point: while stocking practices may be dangerous because they erode the endemic gene pool, they do increase the genetic diversity and decrease inbreeding. This information has to be taken into account when implementing conservation strategies.

The patterns of nucleotide diversity in Table 1 showed that the values were lower in the Carpione, Atlantica and Mediterranea Island populations (< 0.5 × 10^–3^) and higher in the Mediterranea Mainland population (1.12 × 10^–3^). The nucleotide diversity measured by considering the whole sample as a single population was 1.45 × 10^–3^. These recorded values confirm the low levels of the genetic diversity in the Italian samples; indeed, the nucleotide diversity coefficients that were recently reported for wild and farmed French brown trout were higher, ranging from 0.8 × 10^–3^ to 2.6 × 10^–3^ [1].

Additional file 9: Table S5 integrates the genetic map of *Salmo trutta* [38] that we anchored on the *Salmo trutta* genome, with the levels of heterozygosity along the genome. Figure S5 (see Additional file 10: Fig. S5) shows the levels of heterozygosity according to recombination rate. In the whole sample, we observed a negative correlation between heterozygosity and recombination rates. In the Atlantic and Carpione groups, we found a positive correlation between heterozygosity and recombination rates but no significant correlation was observed in the remaining populations. Previous studies in the Atlantic lineage of brown trout have reported a positive correlation between nucleotide diversity and recombination rate [38]. Studies in other species (e.g. Drosophila) also showed a positive correlation between these measures [44]. Thus, the observation of a negative correlation between heterozygosity and recombination rate in the full sample is puzzling and may be explained by the observed high levels of population stratification in the whole sample.

### Putative signatures of selection

**Fig. 4:**
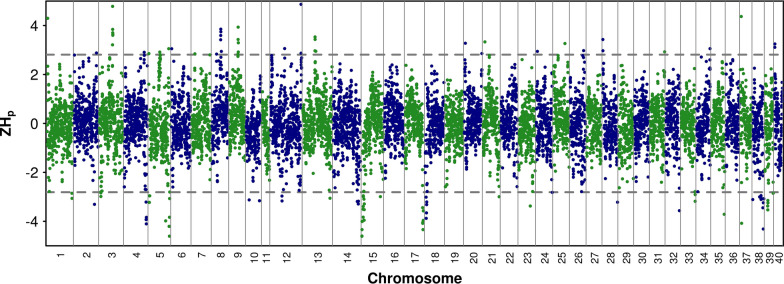
Distribution of ZH_p_ values across the brown trout genome. Horizontal dashed line represents the threshold of ± 2.81 (p < 0.005)

While several of the approaches used here to detect putative signatures of selection are considered to be sound [35–37], the reader should keep in mind that genomic features such as lack of recombination or other mechanisms can generate patterns similar to signatures of selection, which is why the regions we identified in our study are termed *putative* signatures of selection. To identify genomic regions that are putatively under selection, we searched for regions of significantly reduced heterozygosity using the ZH_p_ statistic [36]. Negative ZH_p_ scores indicate an excess of homozygotes and suggest negative or positive selection; positive ZH_p_ scores indicate an excess of heterozygotes and may arise due to balancing selection. The analysis was conducted by treating all the animals as a single population, to search for regions under selection that are shared between lineages. Figure 4 shows the genome-wide distribution of the ZH_p_ scores. Several loci with a ZH_p_ score exceeding the threshold of +2.81 or − 2.81 (p < 0.005) were identified.

Details of the 17 windows that we identified as putatively under selection for either lack or excess of heterozygosity are in Additional file 11: Table S6, together with the results of the KEGG enrichment analysis. Regions with a negative ZH_p_ score show an excess of homozygosity and may be due to positive or negative selection, while regions with a positive ZH_p_ score show an excess of heterozygosity and may be due to balancing selection. Since the ZH_p_ analysis was carried out on the entire sample, it achieves a higher power for signatures of selection that act similarly on all the populations (i.e. signatures that predate the divergence between populations), although we cannot exclude that strong signatures of selection that affect one or more populations are detected. In addition, an excess of expected heterozygosity in genomic regions in structured samples may be caused by population structure, and does not necessarily reflect selection.

We identified 13 significant regions with a negative ZH_p_ score, ranging from − 4.24 to − 2.92 and consisting on average of 7.5 significant windows with an average score of − 3.5 and an average length of 2.39 Mb. In addition, we identified four regions with a positive significant ZH_p_ score, ranging from 2.86 to 3.75 and consisting on average of 4.5 significant windows, with an average score of 3.4 and an average length of 1.85 Mb. As expected, heterozygosity was lower in regions with a negative ZH_p_ (0.09 ± 0.025) than in regions with a positive ZH_p_ score (0.279 ± 0.008); as a comparison the genome-wide average heterozygosity was 0.19 ± 0.165.

We explored patterns of recombination rates around the putative selected regions. Recombination rates in putative regions under selection did not differ significantly from recombination rates averaged across the whole chromosome in which the regions were located. The average recombination rate in the putative selected regions was 0.61 cM/Mb ± 0.49 when including a 1-Mb region up- and down-stream and 0.77 cM/Mb ± 0.57 when including a 5-Mb region up- and down-stream. The average recombination rate across whole chromosomes was 0.7 cM/Mb ± 0.3. However, in two cases, we identified regions under selection that had estimates of recombination rate lower than the chromosome-wide rate. The most striking region was between 27 and 28.6 Mb on chromosome 8 that had a recombination rate of 0.243 cM/Mb compared to the recombination rate of 0.8 cM/Mb averaged across the whole chromosome.

One striking characteristic of the KEGG enriched terms for the windows with a negative ZH_p_ score is the abundance of terms related to infection and diseases; all the significant windows showing enrichment of a KEGG class were enriched for at least one class related to viral infectious diseases (see Additional file 11: Table S6). Among the windows with positive ZH_p_ scores, no window showed enrichment for infectious viral diseases. The ZH_p_ analysis was also performed on each of the populations to identify which populations drive the selection signal (Additional file 11: Table S6, column “Pop”), and on samples stratified into wild and farmed individuals, to assess if one of the conditions was driving most of the signals (see Additional file 11: Table S6, column “Farm.river”), but no outstanding driver of selection signals was found. However, some regions identified in the whole sample overlapped with regions identified in the separate populations, or in samples composed only of farmed or wild fishes. These may represent regions in which selection acted preferentially on a subset of our sample and was also strong enough to be detected in the whole sample. For example, the region between 6.4 and 8.4 M on chromosome 15 overlapped with regions identified in each of the Atlantic, Marmorata and Mediterranea Mainland populations (see Additional file 11: Table S6, column “Pop”), and with a region identified when analyzing only farmed animals (see Additional file 11: Table S6, column “Farm.river”). The results obtained when the population was stratified by sample origin (Farm or River) and by population are in Additional file 12: Fig. S6 and Additional file 13: Fig. S7, respectively.

We also repeated the analysis by excluding the admixed individuals (see Additional file 11: Table S6, column “Q95”) and confirmed 14 of the 17 windows identified using the whole sample; interestingly, the three windows that were not confirmed were among those that were the shortest and among those that, according to our simulations, were most likely to be false positives, consisting of three or four SNPs, with an attributed *p*-value of 0.007 and 0.0004, respectively. Only the region on chromosome 13 overlapped with windows detected by another approach (IBD) (see Additional file 11: Table S6, column “Common”).

**Fig. 5:**
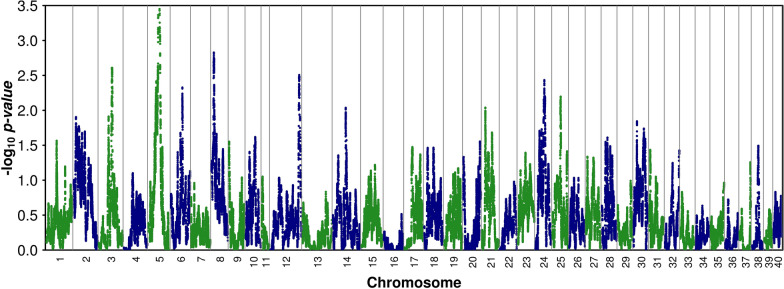
Distribution of the –log_10_ p-values for the haplotype-based analysis of selection across the brown trout genome

To extend the search for selective sweeps, we performed a haplotype-based approach that measures the difference in haplotype frequency between populations while accounting for the relationship structure that exists between the populations, using HapFLK [36]. HapFLK is an extension of the Lewontin and Krakauer statistics, based on F_ST_; HapFLK detects signatures of selection that have led to differences in haplotype frequencies between populations, and thus identifies selective pressures that have caused differentiation between populations. Figure 5 shows the results of the haplotype-based analysis of positive selection from our data. In total, we identified 30 significant regions that had an average length of 1 Mb, and consisted on average of 50 SNPs (see Additional file 14: Table S7). The KEGG terms showing enrichment in the significant windows included ‘Genetic information processing’, ‘Environmental information processing’ and ‘Metabolism’. Three of the 30 regions overlapped with regions identified with the IBD approach. Only six regions overlapped with regions identified in the analysis comparing farmed vs wild individuals, and only seven overlapped with regions identified when admixed individuals were excluded from the analysis. These results are in contrast with those based on the ZH_p_ score, for which most of the windows were confirmed when changing group definition. Such a difference is partly expected, since HapFLK uses differentiation between groups of populations to identify regions under selection in one population, and thus, changing group definition may modify the regions that show different patterns between groups. To go further, we investigated in more detail why the putative regions under selection identified in the whole sample were not detected in the analysis comparing farmed vs. wild individuals. A first group of windows was located on chromosome 5, approximately from 22.8 to 23.2 Mb (see Additional file 14: Table S7), and most of these did not overlap with any of the windows obtained in the comparison between wild vs farmed individuals, although among the latter, there was one significant window located approximately from 23.7 to 25 Mb. Thus, it is possible that, in this instance, the two approaches identified the same putative selection signal, but at slightly different positions. Then, we investigated the windows located on chromosome 18 and noticed that for some of the corresponding detected windows, the *p*-value approached, but never reached, significance. In this case, changing the group definition reduced the power of detection, which suggests that the selection signal observed on chromosome 18 may not be related to domestication in our sample. Finally, one window was detected on chromosome 30 and one on chromosome 32, and in both cases, the *p*-value observed by contrasting wild vs farmed individuals was below significance; this may again indicate a reduction in the power of detection. The levels of heterozygosity in the putative regions under selection were slightly higher than the genomic average heterozygosity (0.229 ± 0.049 vs 0.19 ± 0.165).

The recombination rate estimated for the putative regions under selection (0.4 ± 0.24 cM/Mb when including 1 Mb up- and down-stream of the region) was lower than the average chromosomal recombination rate (0.57 ± 0.17), which suggests that at least part of the signal is due to hitchhiking.

Finally, we used the kinship coefficients estimated via IBD levels to identify regions that are shared between populations as proxies of signatures of selection; locally, high levels of IBD between individuals belonging to the different populations should enable the detection of any kind of selection, regardless of whether it is positive, negative or balancing. Table S8 (see Additional file 15: Table S8) shows the putative regions under selection according to this approach. Only four regions were retained as showing a significant excess of IBD compared to background levels. All the regions identified by IBD overlapped with regions detected by the other approaches: three overlapped with windows identified by the HapFLK approach and one with windows detected by the ZH_p_ approach. In addition, all four windows were also identified in the analysis comparing samples from farms and samples from river (see Additional file 15: Table S8), and when admixed individuals were excluded. The average level of heterozygosity was 0.204 ± 0.01, which is comparable to the genome-wide heterozygosity.

The recombination rate measured in the selected regions was lower than average: 0.12 ± 0.04 cM/Mb when including 1 Mb up- and down-stream, and 0.2 ± 0.08 cM/Mb when including 5 Mb up- and down-stream, compared with the average value of 0.46 ± 0.07 cM/Mb for the corresponding chromosomes. This suggests that part of the detected signal is possibly due to genetic hitchhiking.

Among the enriched terms listed in these regions, the regions on chromosomes 2, 18 and 32 are enriched for the KEGG classes related to infectious diseases, with those on chromosomes 2 and 32 related to bacterial infection, and that on chromosome 18 related to viral disease. No enrichment for genes involved in infectious diseases was observed in the region on chromosome 13.

Because in this study, the analysis was based on the complete sample of individuals, the highest power was achieved when selection acted in all the populations, which may be the case for events that preceded the separation of populations. However, selection causing a local increase of IBD between a pair of populations can also be detected, which may be the case for three of the four identified windows that were also detected by HapFLK, and are therefore expected to differ between groups of populations.

It should be noted that some limitations apply to our study, i.e. due to sampling difficulties, some samples were collected directly from rivers, while others were collected on fish farms, and also different lineages have different sample sizes (see Additional file 1: Table S1). This makes it challenging to formulate specific hypotheses linking selection with population history.

## Conclusions

Our results show that the studied sample is divided into five distinct lineages: Marmoratus, Carpione, Atlantic, Mediterranea Mainland and Mediterranea Island. All lineages were genetically well separated, and high levels of admixture were only detected between Atlantic and Mediterranea Mainland. All lineages had low genetic diversity and high levels of inbreeding and kinship. Mediterranea Mainland was the lineage showing the lower levels of inbreeding and kinship, and the higher genetic diversity, probably because of gene flow from the Atlantic lineage. Analysis of the regions that are putatively under selection revealed an enrichment of KEGG terms related to viral infections in regions with negative ZH_p_ scores. In general, several putatively regions under selection are implied in resistance to diseases; this information may be of interest for fostering future studies on genetic susceptibility to diseases, and can lead to improved breeding programs.

## Supplementary Information

**Additional file 1: Table S1.** Sample description. Sample metadata, including sample name and origin and, for samples passing quality control, Sequence Read archive Run (SRR) accession number, genotype assignment to each population and MaxQ.

**Additional file 2: Table S2.** Number of SNPs per chromosome. This table shows the chromosome names according to RefSeq and INSDC, chromosome number, number of SNPs per chromosome, and chromosome length in Mb.

**Additional file 3: Table S3.** Number of SNPs retained according to filtering thresholds. This table shows the number of SNPs retained for further analyses after applying different filters for MAF and LD.

**Additional file 4: Figure S1.** Admixture cross-validation error distribution as a function of K.

**Additional file 5: Figure S2.** Population structure according to the PCA analysis.

**Additional file 6: Table S4.** Results of the identification of species hybrids using NewHybrids in admixed individuals**.** This table shows the probability of different ancestry scenarios for admixed individuals.

**Additional file 7: Figure S3.** Distribution of kinship (A) and inbreeding coefficients (B) with different MAF thresholds. The four panels were obtained using four MAF thresholds. (a) MAF > 0.05, (b) MAF > 0.1, (c) MAF > 0.2, (d) MAF > 0.25. MI: Mediterranea Island, AT: Atlantic, MM: Mediterranea Mainland, MA: Marmoratus, CA: Garda’s Carpione

**Additional file 8: Figure S4.** Distribution of kinship (a) and inbreeding coefficients (b) separating the Mediterranea Mainland sub-populations. MI: Mediterranea Island, AT: Atlantic, Bif: Biferno (Mediterranea Mainland), Sec: Secchia (Mediterranea Mainland), Ser: Serchio (Mediterranea Mainland), Fib: Fibreno (Mediterranea Mainland), Vol: Volturno (Mediterranea Mainland), MA: Marmoratus, CA: Garda’s Carpione

**Additional file 9: Table S5.** Anchored linkage map of *Salmo trutta*. Linkage map of *Salmo trutta*, anchored on the *Salmo salar* genome as in the original study [38], and on the *Salmo trutta* genome as performed in the present study. The last six columns report the mean heterozygosity for the whole dataset and for each of the five lineages, respectively.

**Additional file 10: Figure S5.** Heterozygosity level according to the recombination rate in the complete dataset and in each population. R^2^ and the corresponding p-value are shown in the top right corner of each graph. Total: whole dataset, AT: Atlantic, CA: Garda’s Carpione, MA: Marmoratus, MM: Mediterranea Mainland, MI: Mediterranea Island

**Additional file 11: Table S6.** KEGG enrichment in putative selected regions according to ZH_p_. Genomic regions showing the strongest signatures of selection according to ZH_p_, together with the KEGG classes and pathways showing significant enrichment in the regions.

**Additional file 12: Figure S6.** Distribution of ZH_p_ values across the brown trout genome for the farmed subset (A) and for the wild subset (B). Horizontal dashed line represents the threshold of ± 2.81 (p < 0.005)

**Additional file 13: Figure S7.** Distribution of ZH_p_ values across the brown trout genome, separately for each of the five populations. MA: Marmoratus, CA: Garda’s Carpione, AT: Atlantic, MM: Mediterranea Mainland, MI: Mediterranea Island. Horizontal dashed line represents the threshold of ± 2.81 (p < 0.005)

**Additional file 14: Table S7.** KEGG enrichment in putative selected regions according to HapFLK. Genomic regions showing the strongest signatures of selection according to HapFLK, together with the KEGG classes and pathways showing significant enrichment in the regions.

**Additional file 15: Table S8. **KEGG enrichment in putative selected regions according to IBD level. Genomic regions showing the highest IBD level, together with the KEGG classes and pathways showing significant enrichment in the regions.

## Data Availability

The sequencing reads used in this study are available in Sequence Read Archive, under the accession PRJNA663991 (https://www.ncbi.nlm.nih.gov/sra/PRJNA663991). The VCF file with the genotypes used in this work is available in the figshare repository, with the identifier https://doi.org/10.6084/m9.figshare.12999725. Scripts and functions used in the present work are released on github under the GNU General Public License v3.0: (https://github.com/genomeud/GenSal).
