# Supplementary figures and images for: ddRAD-seq reveals the genetic structure and detects signals of selection in Italian brown trout
Source: Genet Sel Evol. 2022 Jan 31;54:8. doi: 10.1186/s12711-022-00698-7 (PMC8805291; doi:10.1186/s12711-022-00698-7)

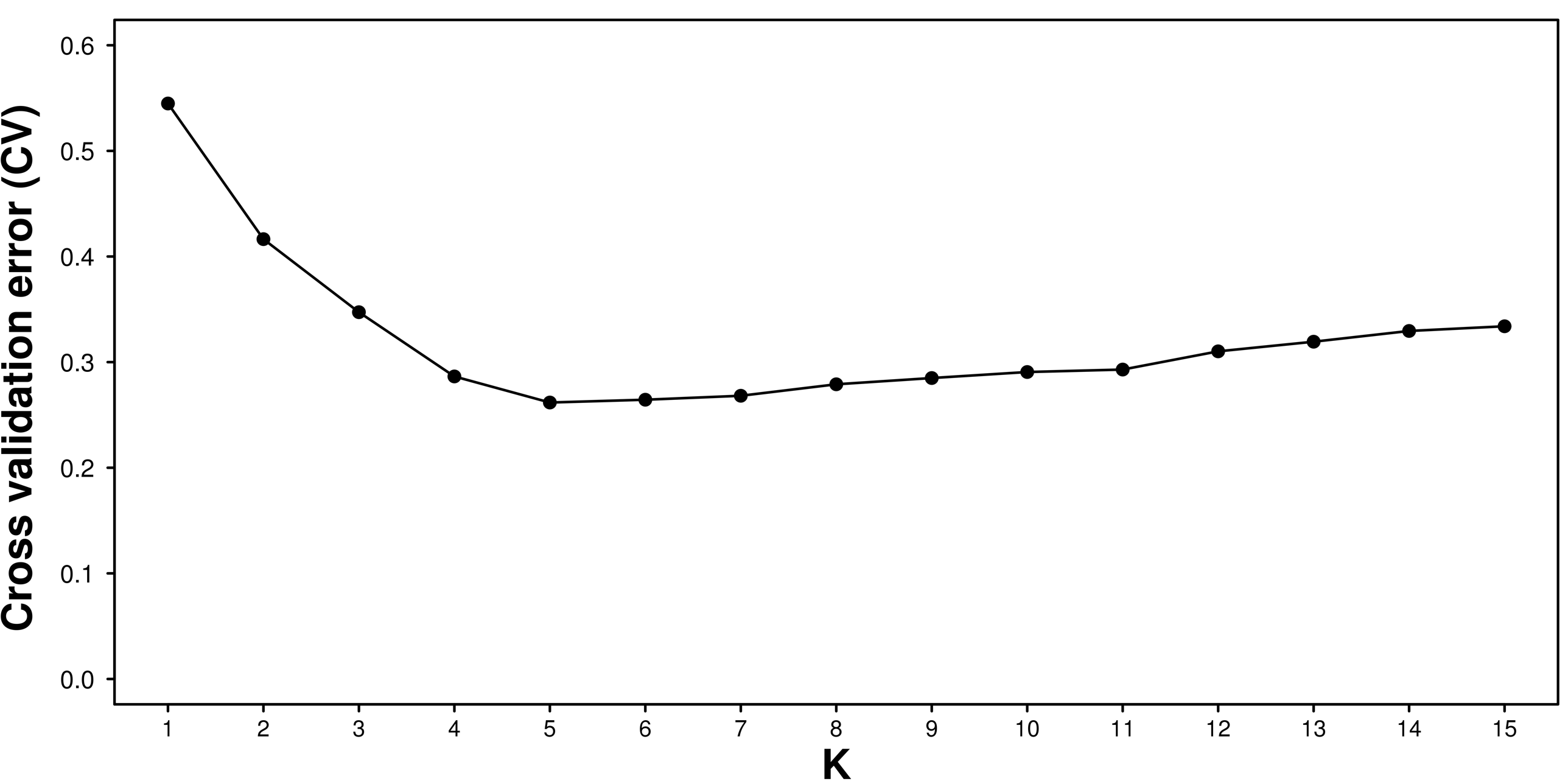

Supplement: Supplementary file 4 — Additional file 4: Figure S1. Admixture cross-validation error distribution as a function of K. [file 12711_2022_698_MOESM4_ESM.jpg]

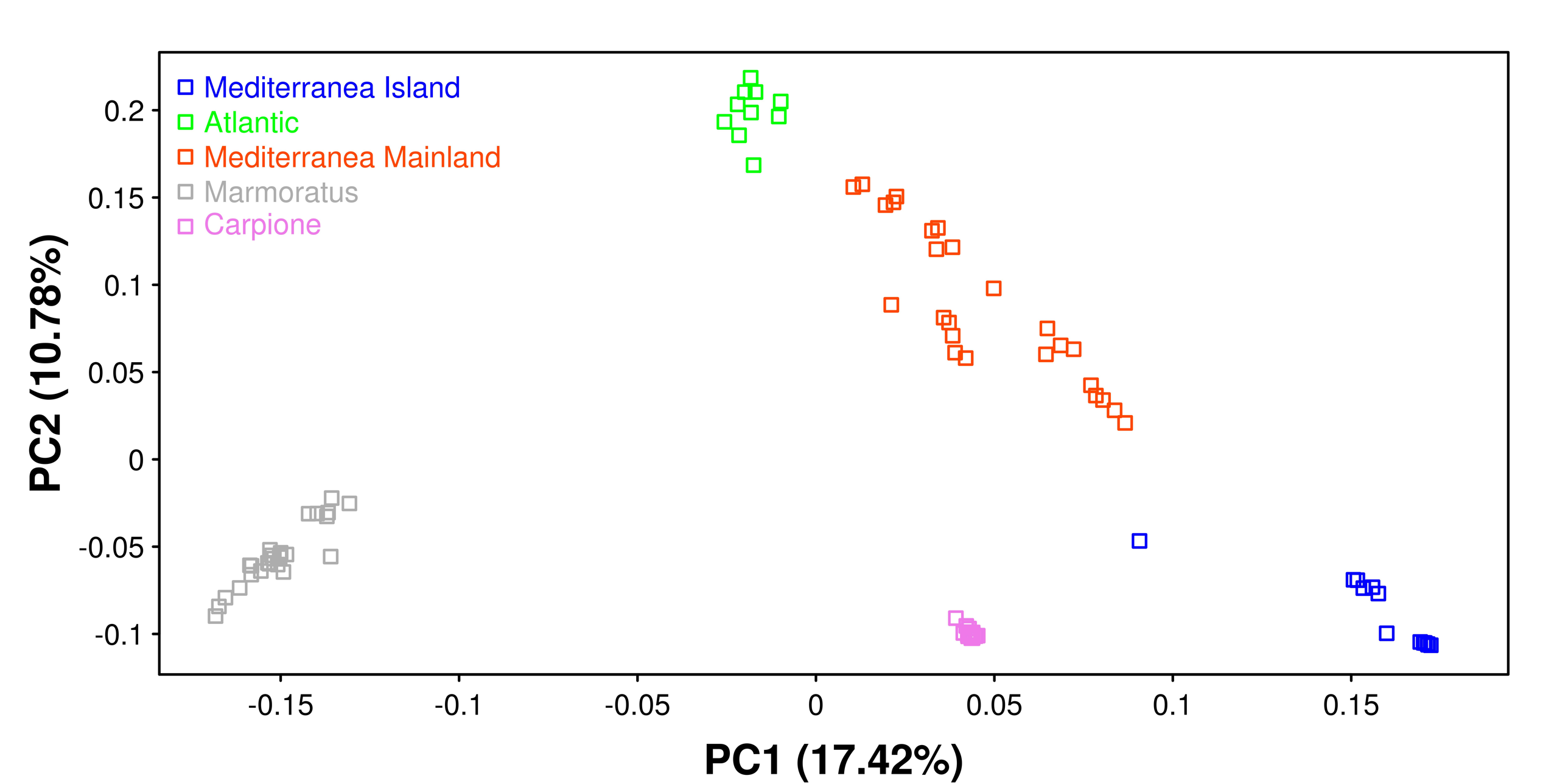

Supplement: Supplementary file 5 — Additional file 5: Figure S2. Population structure according to the PCA analysis. [file 12711_2022_698_MOESM5_ESM.jpg]

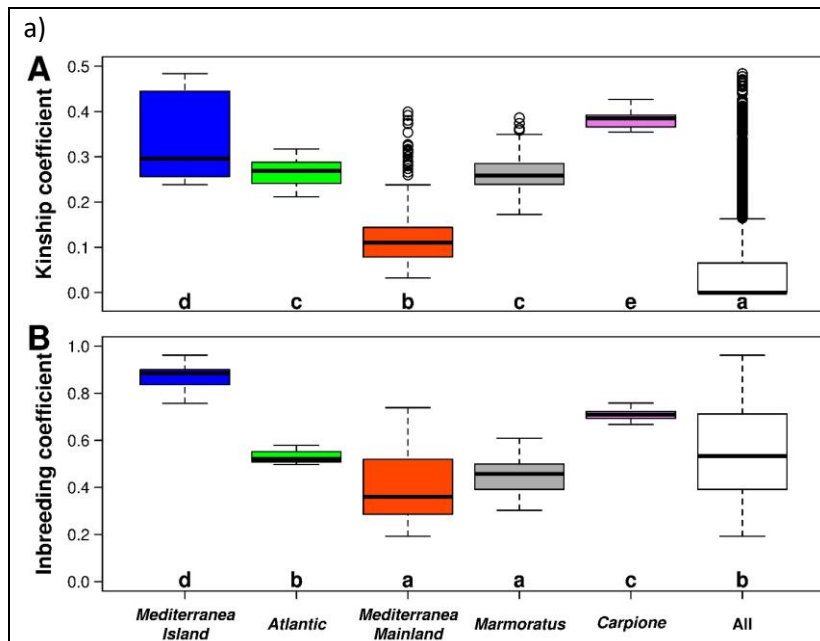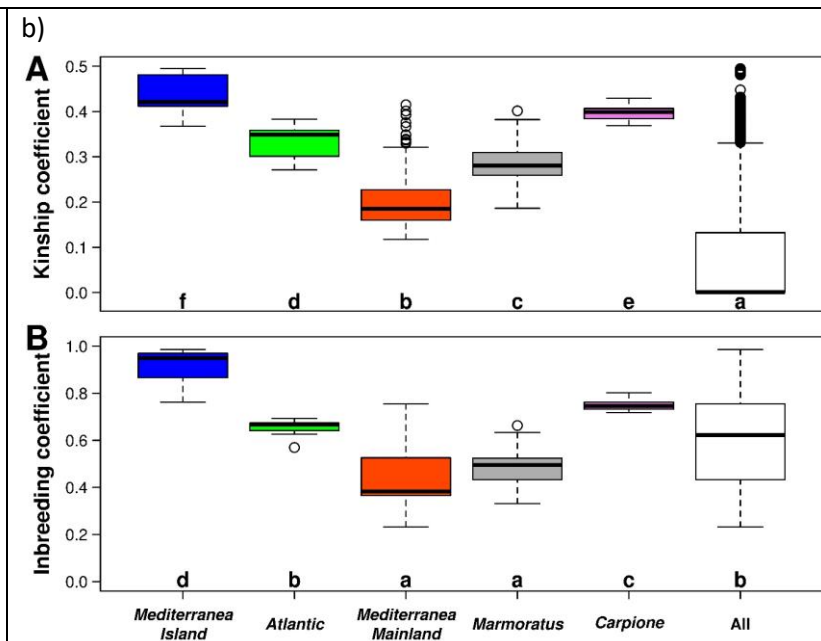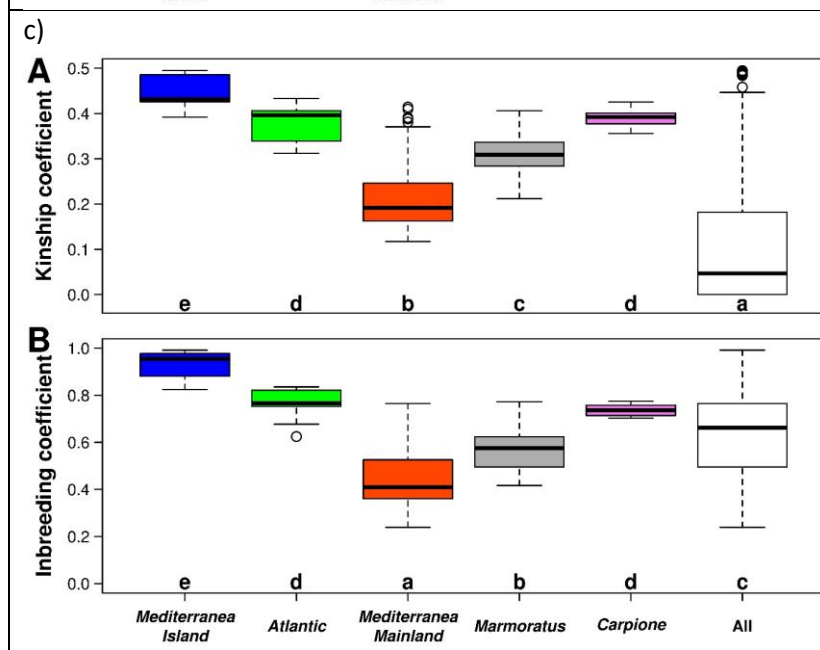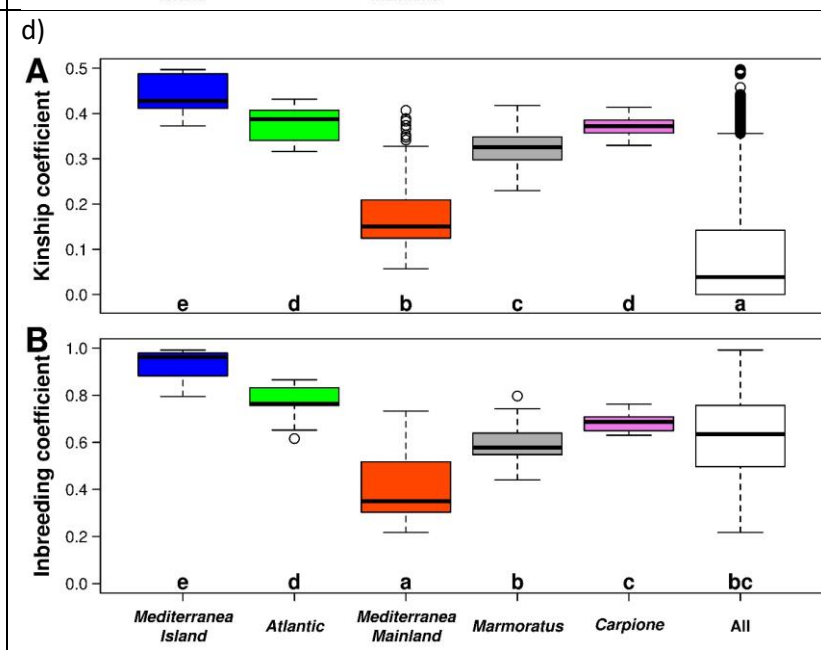

Supplement: Supplementary file 7 — Additional file 7: Figure S3. Distribution of kinship (A) and inbreeding coefficients (B) with different MAF thresholds. The four panels were obtained using four MAF thresholds. (a) MAF > 0.05, (b) MAF > 0.1, (c) MAF > 0.2, (d) MAF > 0.25. MI: Mediterranea Island, AT: Atlantic, MM: Mediterranea Mainland, MA: Marmoratus, CA: Garda’s Carpione [file 12711_2022_698_MOESM7_ESM.pdf]

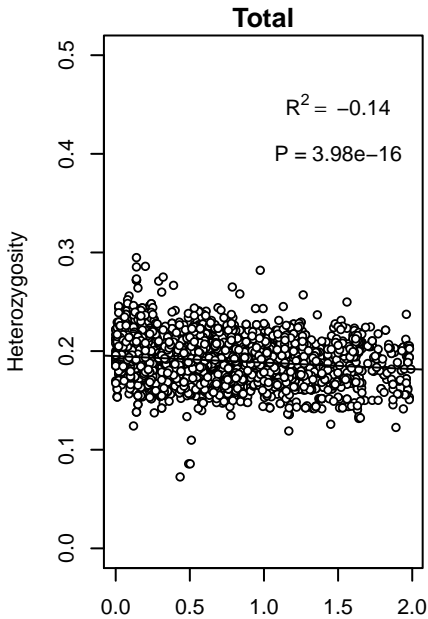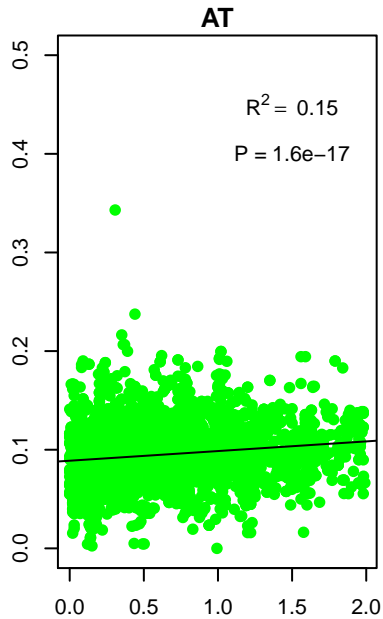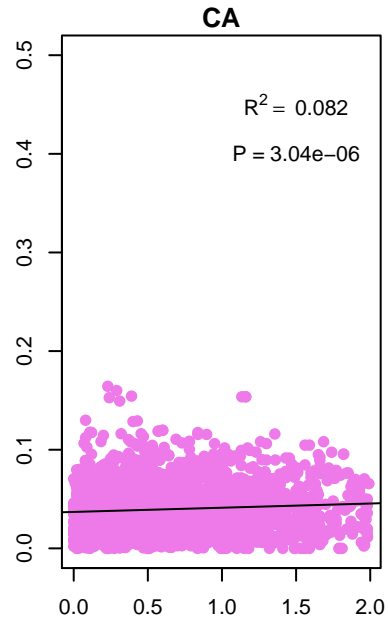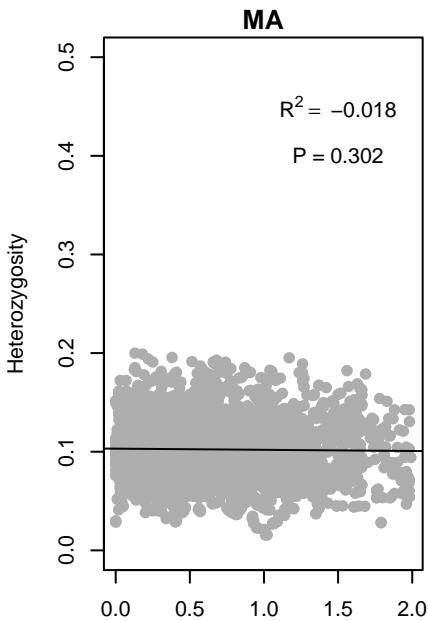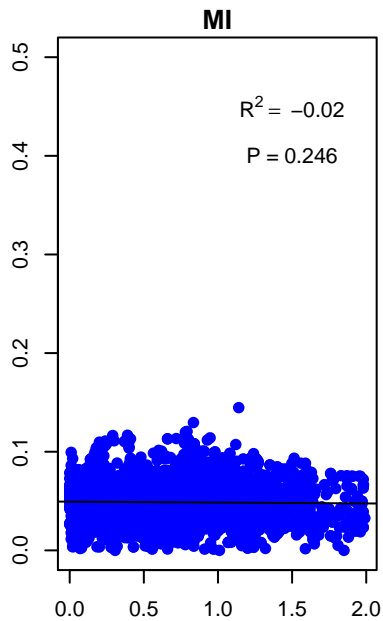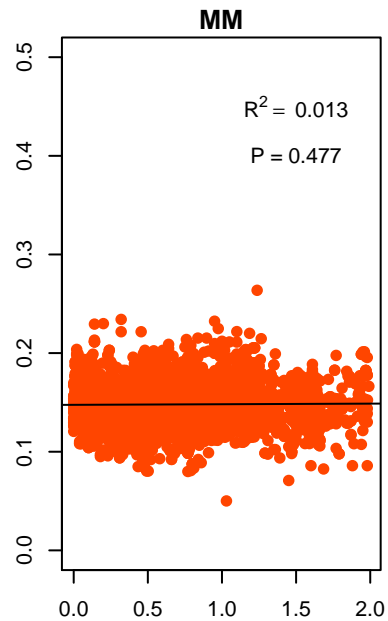

Supplement: Supplementary file 10 — Additional file 10: Figure S5. Heterozygosity level according to the recombination rate in the complete dataset and in each population. R2 and the corresponding p-value are shown in the top right corner of each graph. Total: whole dataset, AT: Atlantic, CA: Garda’s Carpione, MA: Marmoratus, MM: Mediterranea Mainland, MI: Mediterranea Island [file 12711_2022_698_MOESM10_ESM.pdf]

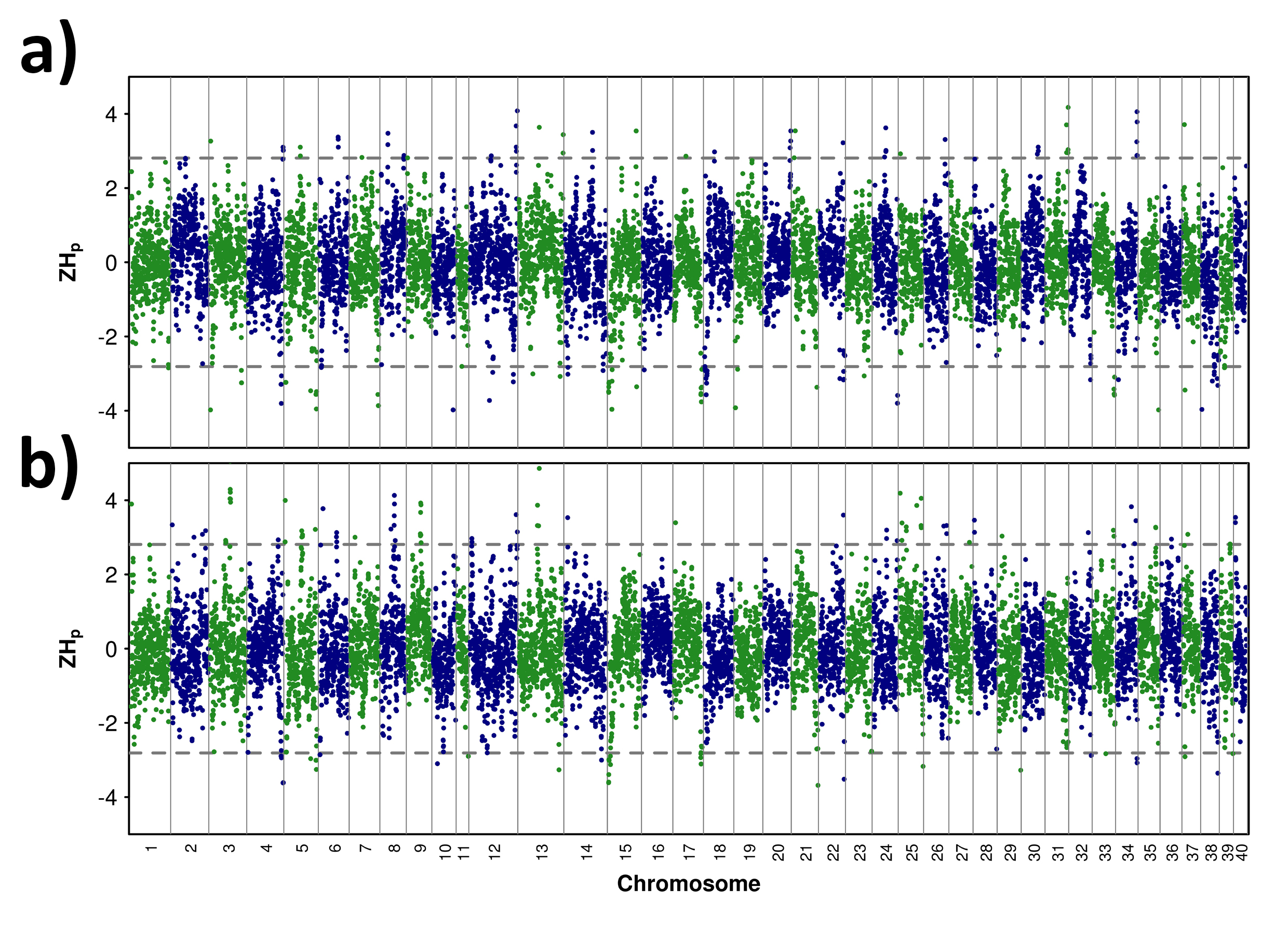

Supplement: Supplementary file 12 — Additional file 12: Figure S6. Distribution of ZHp values across the brown trout genome for the farmed subset (A) and for the wild subset (B). Horizontal dashed line represents the threshold of ± 2.81 (p < 0.005) [file 12711_2022_698_MOESM12_ESM.jpg]

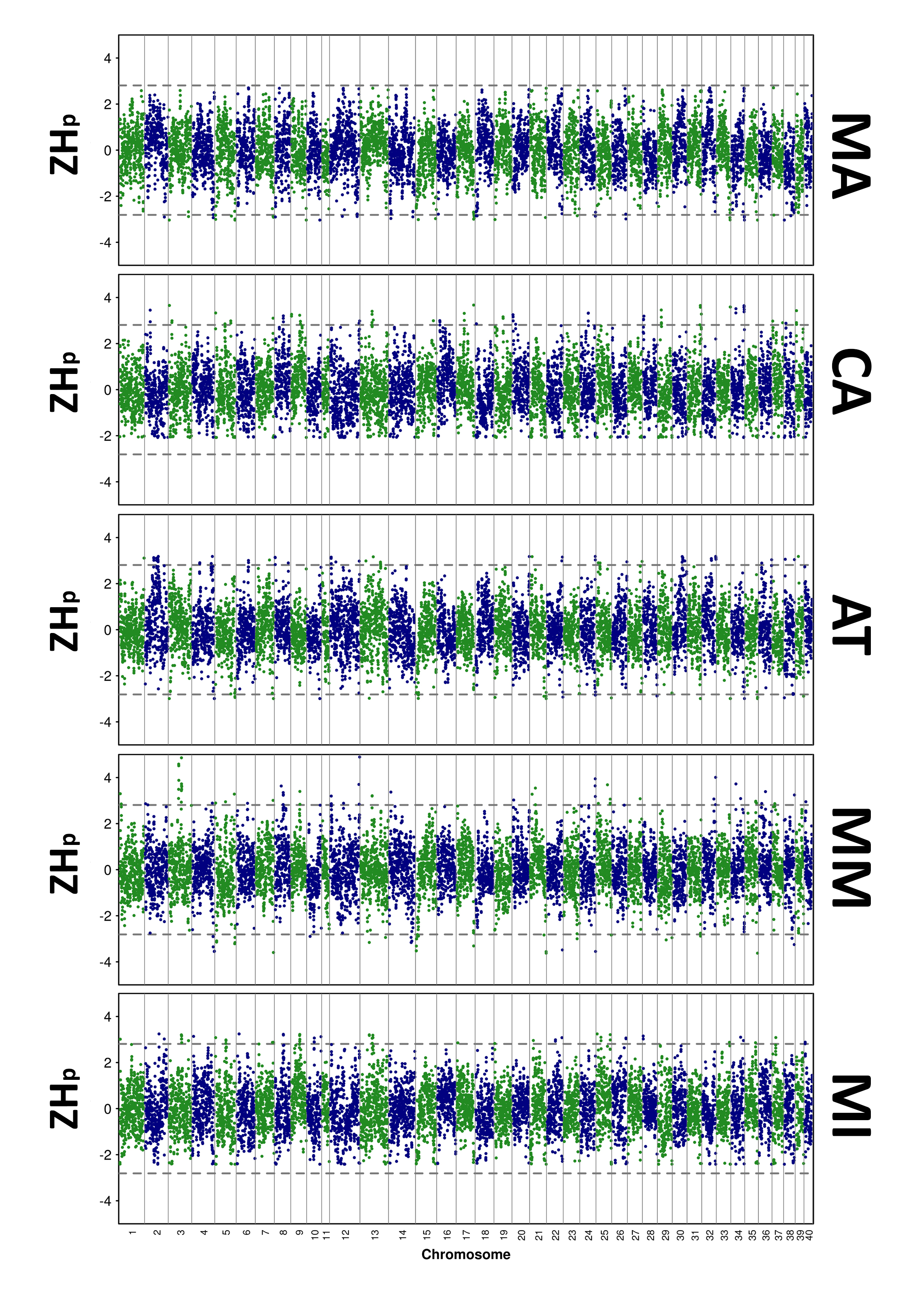

Supplement: Supplementary file 13 — Additional file 13: Figure S7. Distribution of ZHp values across the brown trout genome, separately for each of the five populations. MA: Marmoratus, CA: Garda’s Carpione, AT: Atlantic, MM: Mediterranea Mainland, MI: Mediterranea Island. Horizontal dashed line represents the threshold of ± 2.81 (p < 0.005) [file 12711_2022_698_MOESM13_ESM.jpg]
